# Applications of Bone Morphogenetic Proteins in Dentistry: A Bibliometric Analysis

**DOI:** 10.1155/2020/5971268

**Published:** 2020-10-24

**Authors:** Paras Ahmad, Elena Della Bella, Martin J. Stoddart

**Affiliations:** AO Research Institute Davos, 7270 Davos Platz, Switzerland

## Abstract

**Background:**

Many articles on bone morphogenetic proteins (BMPs) have been published. Bibliometric analysis is helpful to determine the most influential studies in a specific field. This bibliometric analysis is aimed at identifying and analyzing the top 50 most-cited articles on the dental applications of BMPs.

**Methods:**

An electronic search was conducted using the Web of Science (WoS) “All Databases” without any restriction of language, study design, or publication year. Of 1341 publications, the top 50 were included based on their citation count. After downloading the full texts, their bibliometric data including publication title, authorship, citation count, current citation index 2019, citation density, year of publication, country and institution of origin, journal of publication, type of BMP, study design, evidence level of publication, and keywords were extracted and analyzed.

**Results:**

The citation counts for the top 50 publications ranged from 81 to 557 (median 113.5). The most prolific year was 1997 (*n* = 7). Wikesjö UM (*n* = 12) and Wozney JM (*n* = 11) were the major contributors in this study. Most of the articles were generated primarily from the USA (*n* = 24), with Loma Linda University Medical Center, USA being the most prolific institution (*n* = 5). Majority of the articles were published in the *Clinical Oral Implants Research* and *Journal of Periodontology*, with nine publications each. Most of the publications were animal studies (*n* = 30) and focused on BMP-2 (*n* = 39). Most of the articles were within evidence level V (*n* = 36). The most frequently used keyword in the top articles was “bone regeneration” (*n* = 23).

**Conclusion:**

The present study presents insights into the past and recent trends in the applications of BMPs in dentistry. A statistically significant association was observed between citation count, citation density, and age of publication.

## 1. Introduction

As the inducers of ectopic bone formation, bone morphogenetic proteins (BMPs) were first discovered by the American orthopedic surgeon Marshall R. Urist, who reported his results in the semen samples [1]. More than 30 BMPs have been identified in different organisms to date, including mammals, sea urchin, *C*. *elegans*, *Xenopus*, and *Drosophila* [1, 2], and now, they are acknowledged in their role as pleiotropic growth factors found both in vertebrates and in invertebrates [2]. BMPs are members of the transforming growth factor beta (TGF-*β*) superfamily (with the exception of BMP-1, which is a metalloendopeptidase) and are known to play their part in a multitude of processes during development and homeostasis. They are involved in several biological activities such as morphogenesis, cell differentiation, healing, and regeneration [3–8]. They are also known to be involved in chondrogenesis and osteogenesis, and they play a vital role in fracture healing and embryonic development [9–12]. Furthermore, they are involved in the maintenance of various vital organs including the kidney, cartilage, muscle, bone, and blood vessels [10].

The osteoinductive functions are displayed by only a small number of BMPs, such as BMP-2, BMP-4, BMP-6, BMP-7, and BMP-14 [11, 13–17], leading to the suggestion that they should be renamed as Body Morphogenetic Proteins. BMP-2, BMP-4, and BMP-7 have been reported to stimulate *in vivo*, *in vitro*, and *de novo* bone formation in several animal models [13, 14, 16, 18]. The osteoinductive capacity of BMPs has led to their application as therapeutic agents for the generation of new bone in order to treat skeletal injuries [19]. BMPs have been approved for clinical use in vertebral arthrodesis [20], nonunions [21], and open fractures of long bones [22] after their definite osteoinductive ability was confirmed by several preclinical and clinical studies. More recently, questions have been raised about their safety [23]. However, the utilization of BMPs is not limited to osteogenic regeneration; there are other research areas including tooth conservation and periodontal regeneration procedures [24–29]. In dentistry, BMPs, particularly BMP-2, are being widely used in many procedures such as alveolar bone regeneration [8, 13–15, 17, 30], sinus lift augmentation [3, 5, 31], dental implants [16, 32], and periodontal [33–37] and dental regeneration [24–29].

Scientists, funding agencies, and research organizations necessitate metrics to determine the impact of research. The impact factor and citations in peer-reviewed publications are a widely recognized measure of scientific impact [38]. Over the past few years, the interest of bibliometric analysis in quantitative analysis for a given field and obtaining statistics to assess the contribution of scientific article to the progress of knowledge escalated, particularly in medical [39–43] and dental fields [44–48]. Citation analysis is the most frequently used method in bibliometrics. Highly cited studies indicate the research interests of certain periods, and high-frequency keywords reflect the hot topics within research [49]. In 1977, Dr. Eugene Garfield, founder of the Institute for Scientific Information (ISI), proposed the term “citation classics” that is a bibliometric concept. Its purpose was the identification as well as acknowledgment of frequently cited research of authors and their peers that would consequently encourage the respective work and its impact on the specialty [50]. A scientific paper having received more than 400 citations is believed by some to be a “classic” article [45]; however, based on the field of research, an article having secured 100 or more citations can also be considered a “classic” [44, 51, 52].

## 2. Methods

### 2.1. Protocol

This study followed the Preferred Reporting Items for Systematic Reviews and Meta-Analyses (PRISMA) [53]. This analysis was exempt from the institutional ethics committee review as it was a bibliometric review.

### 2.2. Search Methodology and Data Source

Articles on the application for BMPs in dentistry were collected from the Dentistry, Oral Surgery, and Medicine Category of the Clarivate Analytics' Web of Science (WoS), considering the “All Databases” section. To prevent daily updating bias, a comprehensive search was performed on a single day, the 1^st^ of March 2020. The title section was searched utilizing the search terms outlined below in the inclusion criteria. There was no restriction of language, study design, and year of publication.

### 2.3. Inclusion and Exclusion Criteria

The inclusion criteria included the presence of “bone morphogenetic protein” OR “bone morphogenic protein” OR “BMP” OR “rhBMP” in the title of the article and publication in the journal belonging to the category of Dentistry, Oral Surgery, and Medicine. The exclusion criteria included the articles which did not include any of the search terms in their title (i.e., “bone morphogenetic protein” OR “bone morphogenic protein” OR “BMP” OR “rhBMP”) and were not published in the journals falling in the category of Dentistry, Oral Surgery, and Medicine (Supplementary Figure 1).

### 2.4. Article Selection

Of 1341 articles, the top 50 most-cited articles were included for bibliometric analysis based on their citation count. Initially, the list was prepared, and the full texts of articles were reviewed, when deemed necessary. After finalizing the complete list of the top 50 most-cited articles, the full texts of the selected studies were downloaded and analyzed to extract their bibliometric data.

### 2.5. Data Extraction

Publication title, authorship, citation count (extracted from WoS and Google Scholar), Current Citation Index (CCI) 2019 (citation received in the preceding year of conducting the study) [54], citation density (citation per annum) [55], year of publication, country and institution of origin, journal of publication, type of BMP, study design and evidence level of publication, and keywords were extracted and analyzed for each selected article. Furthermore, if two or more articles had the same citation count, the article with the higher citation density was ranked higher.

### 2.6. Journal Metrics

Three indicators, i.e., 5-year journal impact factor (http://www.jcr.clarivate.com), CiteScore (http://www.journalmetrics.scopus.com), and Eigenfactor Score (http://www.eigenfactor.org), were used to determine the relative position of journals.

1. Five-year journal impact factor: this indicator represents the citation counts received by a journal, in one year, of the citable papers published in the last 5 years. Its calculation follows the following formula: citations from journal citation report (JCR) year of documents published in the last 5 years, divided by the total number of citable documents [56].
2. CiteScore (CS): this is a newly introduced indicator adopted to assess the impact of journals so that more rigorous results can be obtained. Its calculation follows the following formula: the ratio of citation counts from all items in 1 year to all items published over the past 3 years for a journal [56].
3. Eigenfactor Score (ES): this is regarded as an indicator of the global repercussions or impact of documents published online in JCR. Its calculation is based on the citation counts of items published in the past 5 years in the JCR per annum. It also takes into account which journals have contributed to these citations, so that highly cited journals will impact the network greater than lesser-cited journals; references from one paper to another paper from the same journal are eliminated, so that ES is not biased by journal self-citation [56].

### 2.7. Data and Statistical Analysis

The Visualization of Similarities viewer (VOSviewer) software [57] was used to create collaboration network maps regarding the cooccurrences of all keywords. IBM SPSS Statistics version 24.0 (IBM, Chicago, IL) was used to perform descriptive and bivariate analyses. The Shapiro-Wilk test was used to evaluate the data distribution. Based on normality and distribution of the data, the mean (standard deviation) or median (interquartile range) was calculated. The Kruskal-Wallis test was done to check median differences between the independent groups. *Post hoc* testing was used to evaluate the median differences within each group. The Mann-Kendall trend test was utilized to analyze any increase or decrease in the time-dependent trends. To analyze the correlation between the age and publication count of the journal, a Spearman-rank test was conducted. A value of *p* < 0.05 was considered statistically significant. In order to screen out the most important independent variables, simple linear regression was performed.

## 3. Results

### 3.1. Citation Count, Current Citation Index, and Citation Density

Supplementary Table 1 depicts the principal characteristics of the top 50 most-cited articles. The citation count of the top 50 articles varied from 81 to 557 (median, 113.5), with a total citation count of 6,847. The most cited article, with a total of 557 citations, was titled “Bone Morphogenetic Protein” (Urist and Strates, 1971) [1] and was published in the *Journal of Dental Research*. Its citation density is 11.60, with a current citation index (CCI) of 15. The second most cited article, with a total of 270 citations, was titled “Randomized Study Evaluating Recombinant Human Bone Morphogenetic Protein-2 for Extraction Socket Augmentation” [58] and was published in the *Journal of Periodontology*. Its citation density is 19.29, with a CCI of 22. The third most cited article, with a total of 261 citations, was titled “Dentin Regeneration by Dental Pulp Stem Cell Therapy with Recombinant Human Bone Morphogenetic Protein 2” [59] and was published in the *Journal of Dental Research*. Its citation density is 17.40, with a CCI of 11.

According to the CCI 2019, the top-ranked article was the randomized controlled trial (RCT) published in 2005, securing 22 citations [58]. The second-ranked article was also the RCT written by Jung et al., in 2003, with 16 citations [60]. The third-ranked article was the animal study written by Urist and Strates in 1971, with 15 new citations [1]. As per citation density, again, the RCT by Fiorellini et al. has the highest score [58]. The article with the second highest citation density, i.e., 18.07, was a clinical trial written by Boyne et al., in 2005 [61]. The third ranked article was the RCT conducted by Triplett and coworkers, in 2009, with a citation density of 17.60 [62].

**Figure 1:**
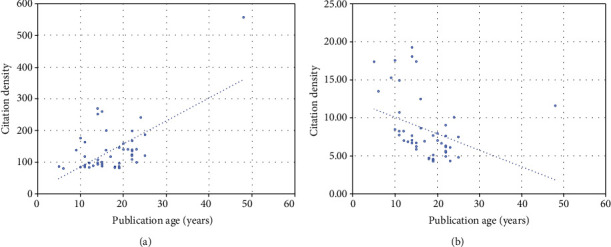
Association of (a) citation count with age of publication and (b) citation density with age of publication.

The distribution of data regarding citation count, citation density, and article age were not normal (Shapiro-Wilk test; *p* < 0.05). A significant trend towards a higher citation count with article age was observed (*r* = 0.189, *p* = 0.042) (Figure 1(a)). However, a significantly negative trend towards an increased citation density with the age of publication was observed (*r* = −0.482, *p* = 0.039) (Figure 1(b)).

### 3.2. Distribution by Year

**Figure 2:**
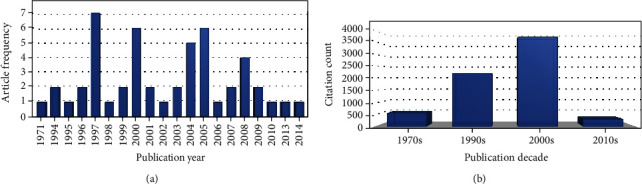
(a) Citation analysis of the top 100 most-cited articles over the years and (b) the decades.

The top 50 most-cited studies were published between 1971 [1] and 2014 [63] (Figure 2(a)). The most productive year in terms of publications was 1997 (*n* = 7), followed by 2000 and 2005, with 6 publications each. The year with the most citations was 1997, with 996 citations, followed by 2005 and 2004, with 931 and 632 citations, respectively. The decade with the most publications (*n* = 31) and citations (*n* = 3,757) was 2000 (Figure 2(b)).

### 3.3. Contribution of Authors

**Figure 3:**
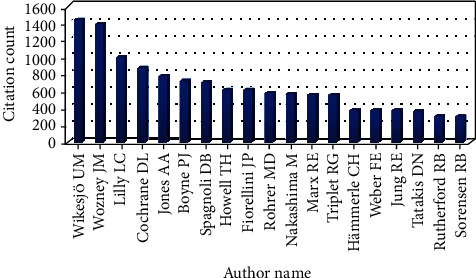
Citation analysis of the authors who contributed to the top 50 most-cited articles.

A total of 177 different authors contributed to the list of top-cited articles. Many of the articles (*n* = 38) had between one and six authors, but publications with more than five authors were the most common (*n* = 15). The majority of the contributions were made by Wikesjö UM (*n* = 12, 1430 citations), followed by Wozney JM (*n* = 11, 1376), Lilly LC (*n* = 5, 992), Cochrane DL (*n* = 5, 866), Rohrer MD (*n* = 5, 578), Jones AA (*n* = 4, 775), and Boyne PJ (*n* = 4, 717) (Figure 3).

### 3.4. Contribution of Countries and Institutions

**Figure 4:**
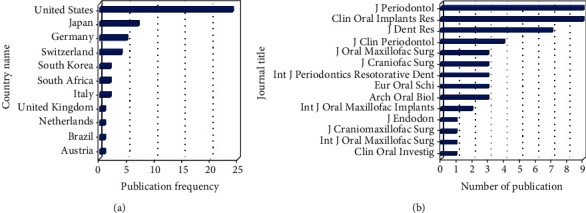
Contributing (a) countries and (b) journals to the top 50 most-cited articles.

The top 50 most-cited publications originated from 11 countries, including Austria, Brazil, Germany, Italy, Japan, Netherlands, South Africa, South Korea, Switzerland, the United Kingdom (UK), and the United States of America (USA) (Figure 4(a)). According to the number of publications, most of the articles originated from the USA (*n* = 24, 3948 citations), followed by Japan (*n* = 7, 985), Germany (*n* = 5, 511), Switzerland (*n* = 4, 475), South Korea (*n* = 2, 217), South Africa (*n* = 2, 185), Italy (*n* = 2, 168), Brazil (*n* = 1, 97), Austria (*n* = 1, 88), Netherlands (*n* = 1, 87), and the UK (*n* = 1, 86).

There was a total of 27 institutions with which the corresponding authors were affiliated. The most prolific institution, with 5 publications, was Loma Linda University Medical Center, USA, followed by School of Medicine, University of California Los Angeles (UCLA), USA; Faculty of Dental Sciences, Kyushu University, Japan; Dental School, University of Zurich, Switzerland; School of Dentistry, Temple University, USA; and School of Dentistry, University of Michigan, USA, with 3 publications each.

### 3.5. Journal of Publication

The top 50 most-cited articles were published in both specialized and comprehensive periodicals (*n* = 14) (Figure 4(b) and Table 1). The journals with the greatest number of publications were *Clinical Oral Implants Research* and *Journal of Periodontology*, with nine publications each, followed by the *Journal of Dental Research* (*n* = 7) and *Journal of Clinical Periodontology* (*n* = 4). The *Journal of Dental Research* had the highest citation count (*n* = 1504), followed by the *Journal of Periodontology* (*n* = 1393) and *Clinical Oral Implants Research* (*n* = 970). Journals' impact factors ranged from 0.785 (*Journal of Craniofacial Surgery*) to 5.125 (*Journal of Dental Research*).

A statistically nonsignificant trend (*p* = 0.192) was observed between a journal age and the number of “classic” articles published in that journal. However, a statistically significant trend (*p* < 0.018) was observed between the impact factor of the journal and the number of “classics” published in that journal. According to the simple linear regression analysis, a statistically significant association was observed between self-citation (*p* = 0.025), CiteScore (*p* = 0.036), Eigenfactor Score (*p* = 0.015), and total citation count (Table 2).

### 3.6. Type of BMP

According to the topic of the article, the majority of the topic covered by the top 50 publications was BMP-2 (*n* = 39, 5174 citations), followed by BMP-7 (*n* = 6, 580), BMP-4 (*n* = 2, 308), more than one BMPs (*n* = 2, 697), and BMP-12 (*n* = 1, 88) (Table 3). No statistical significance was observed (*p* = 0.428) while checking the median difference in the citation count per article, among BMP-2 (median: 118; range: 81-270), BMP-7 (87.5, 81-138), BMP-4 (154, 121-187), more than one BMPs (348.5, 140-557), and BMP-12 (88, 88).

### 3.7. Study Design of Publication

The most common study design in the top 50 articles was animal study (*n* = 30, 4132 citations), followed by clinical trial (*n* = 8, 1105), *in vitro* study (*n* = 5, 494), RCT (*n* = 4, 731), case series (*n* = 1, 245), and literature review (*n* = 1, 140) (Table 3). No statistical significance was observed (*p* = 0.516) while checking the median difference in the citation count per article, among animal study (median: 113.5; range: 81-557), clinical trial (116, 87-253), *in vitro* study (88, 86-138), RCT (188, 85-270), case series (112.5, 81-164), and literature review (140, 140).

### 3.8. Evidence Level of Publication

According to the hierarchy of evidence levels (ELs), most of the top cited articles were within evidence level V (*n* = 36), followed by EL III (*n* = 8), EL II (*n* = 4) and EL IV (*n* = 2). Among these ELs, the total citation counts (*r* = −0.382, *p* = 0.128) and the citation density (*r* = 0.108, *p* = 0.633) did not vary significantly.

### 3.9. Keywords

**Figure 5:**
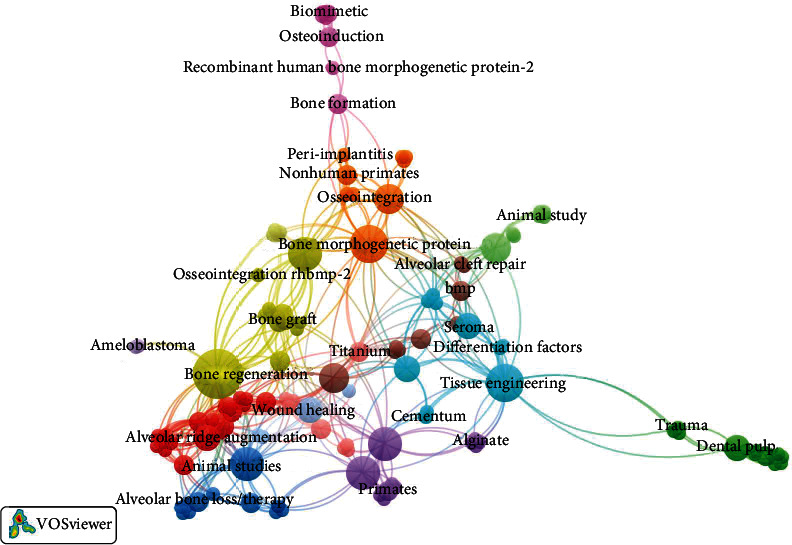
Network analysis of keywords. The size of the node represents the frequency of the keywords, with larger nodes indicating higher frequency.

Out of the top 50 most-cited publications, only 37 articles contained keywords. A total of 182 keywords were identified (Figure 5). The most frequently used keyword was bone regeneration (n =23), followed by bone morphogenetic protein (*n* = 11), animal studies (*n* = 7), tissue engineering (*n* = 6), dental implants (*n* = 6), periodontal regeneration (*n* = 6), protein (*n* = 6), bone morphogenetic protein-2 (*n* = 5), and growth factor (*n* = 5).

## 4. Discussion

To address the nonexistence of bibliometric analyses on the applications of bone morphogenetic proteins (BMPs) in dentistry, this study is aimed at identifying and analyzing the top 50 most-cited papers on BMPs along with its current status of research activity. In general, when a scientific article makes its appearance on the list of most frequently cited articles in its respective discipline, it shows that it has reached a landmark [46]. In theory, the acknowledgment of a research within the scientific area (citation count) and how it influenced the knowledge of a disease and/or its treatment, as well as whether it brought about any new research trends, are the reflection of its quality [64]. Hence, when a publication succeeds to secure its rank in the list of “classic” publications in a particular specialty, it shows that the global clinical and scientific communities recognized both the study and the journal as having made a significant contribution to the specialty [45, 65]. Hence, the findings of the present study not only depict a historical perspective on scientific advancement on BMPs but also display principal trends in research as well as clinical practice.

The precision of bibliometric studies might be negatively influenced by the limitations of the database used. Elsevier's Scopus, Google Scholar, and the Clarivate Analytics' WoS may differ quantitatively or qualitatively regarding the citation count of a research article depending upon its research area [47, 66, 67], journals [68], and year of publication [69]. Moreover, some publications might not be available in all these search engines [54, 66, 70, 71]. For this study, the most potential reason of not using Scopus or Google Scholar database as the benchmark search engine was that the former database does not take into coverage the citation count records of the studies published prior to 1995 [69, 70] and the latter database also includes citations from nonscholarly publications [71]. The findings of the current study reflected a variation in the citation counts of the studies when different search engine was searched. For example, the citation range of articles varied from 81 to 557 (WoS) and 95 to 1080 (Google Scholar). The importance of choosing an appropriate database in scientometry is reflected by this fluctuation in citation counts.

The citation number does not necessarily signify the scientific worth of a publication; however, it does reflect its influence on the progress of the given clinical/research discipline and quantity of the researchers affiliated with that specific specialty. Hence, publications regarding stem cells (927-14,575) [72], cardiology (331-3484) [73], head and neck oncology (628-3205) [74], and respirology (615-2918) [75] are more often cited than publications within dentistry (326-2050) [44], despite all these areas having equal scientific significance.

The year of publication has an undeniable influence on citation a publication would receive. The true influence of a research article can be determined properly at least 20 years after its publication [44, 65, 76]. Hence, older research studies usually achieve more citations than recently published papers, regardless of their influence [77]. This tendency is observed in almost all specialties [39]. The findings of this analysis opposed this trend as 68% of articles were published within the last 20 years. This reflects the rising trends in the BMPs research in recent two decades. For example, the 2^nd^ position in Supplementary Table 1 is achieved by a paper that was published in 2005 [58], securing 270 citations within only 14 years. Similarly, the 3^rd^ and 4^th^ positions are achieved by articles that were published in 2004 [59] and 2005 [61], securing 261 and 253 citations, respectively. This reflects the influence and quality of an article's topic along with its relevance to the clinical practice and research. Concerning the evolution of scientific production, few dental specialties including Periodontology [50] or Implantology [78] have undergone a hike in the quantity of research published in recent years. However, the applications of BMPs in dentistry have followed a different pattern, with increased production up to 2000 followed by a fall. Between 2000 and 2005, research activity took off seeing the greatest number of published studies ever (22/50 articles). The articles published after 2005 also received a notable citation count; however, it is too early to foresee whether these publications would get more citations as time passes.

Regarding the number of authors per publication, the average was 3.54, a lower average than other dental specialties including Periodontology with an average of 5.1 [79] or Implantology with an average of 4.66 [80] and much lower average than medical fields, for example, Cardiology with an average of 10.5 authors per article [81]. However, in the recent years, an increased average number of authors per article have been reported, a progress that corresponds to other medical specialties because of the multidisciplinary nature of much recent research activity [82].

As with several top-cited studies in medical and dental fields, this analysis reported that approximately 50% of the top-cited publications originated from the United States. This significant contribution can be attributed to a larger scientific population, active researchers, and ample financial resources [45, 76, 83–88]. Furthermore, to an unparalleled research work, an increased tendency among authors to cite articles originating from the US has been observed [88, 89]. Several bibliometric studies have reported that authors hailing from institutions of Asia and Africa whether being the first or the corresponding authors made an almost negligible contribution which could be regarded as the “classic article” [76, 83, 88, 90, 91]. An interesting finding of this analysis is that 22% of the most-cited articles were written by authors hailing from Asia (Japan and South Korea) and Africa (South Korea). Furthermore, the European countries such as Austria, Germany, Italy, Netherlands, Switzerland, and the UK, despite their small population, as compared with the US and Asian countries, made considerable contributions (28%). Overall, 27 institutions contributed to the list of 50 most-cited articles; however, one of the major contributions was made by the Los Angeles School of Medicine, The University of California (USA) (*n* = 3). The reason is quite evident from the fact that this is the same institute where BMPs were discovered in the 1960s by Marshall R. Urist. Importantly, a lack of multicenter studies was noteworthy, reflecting a need to escalate the international collaboration.

According to the hierarchy of research evidence, meta-analyses, systematic reviews, and randomized clinical trials (RCTs) provide the highest quality of evidence, whereas literature reviews, case reports, editorials, and opinion papers provide the lowest quality of research evidence [65]. A characteristic feature of the current study was that it included four RCTs [58, 60, 62, 92], but no systematic review or meta-analysis could secure its position in this study. One explanation could be that inadequate time has passed since the publication of more recent systematic reviews or meta-analyses, so it has not yet achieved a significant citation count. Occupying the major bulk of the present study are animal studies (60%) which can be explained by a number of justifications; being biologically similar to humans, animals are susceptible to many of the same health hazards; they have short life cycles, so it is easy to study them throughout their life span. Perhaps, the most important reason is that it would be ethically wrong to intentionally subject humans to health risks for observing the course of the disease [93].

In this analysis, a statistically significant association was found between the number of the top-cited studies published in a journal and the impact factor of that journal. This finding is in accordance with the findings of some bibliometric studies [81, 94–96], but contrary to those of several others [67, 88, 97]. A lack of association indicates that high-quality research might be highly cited, even if it is published in a relatively low impact factor journal [97, 98]. In the present study, majority of the studies (58%) were published in high impact factor journals dedicated to dentistry, oral surgery, and medicine such as the *Journal of Periodontology*, *Journal of Dental Research*, *Journal of Clinical Periodontology*, and *Clinical Oral Implants Research*. Interestingly, after the analysis of all articles published in dentistry journals, it was noteworthy that there were three journals, apart from the aforementioned journals, which published the greatest number of articles on BMPs, i.e., *International Journal of Oral and Maxillofacial Surgery*, *Journal of Craniofacial Surgery*, and *Journal of Oral and Maxillofacial Surgery*. This trend of publication highlights the influence of the impact factor of a journal and the inclination of authors to cite and article that is published in a high impact factor journal within a given specialty.

The journals' ranking based on their impact factor has become a principle consideration when authors decide where to submit their research. The impact factor is corrupted as a proxy for the quality of individual publications [99]. Usually, authors target journals with the highest impact factor instead of journals having the best readers for their article [99]. The evaluation of the scientific influence of journals assessed by bibliometrics is a multidimensional, complex construct, and hence, the utilization of a single bibliometric index is improper to assess, value, and rank journals. Audience should aim beyond the impact factor and evaluate scientific publications individually. Other variables are worth mentioning: each dental specialty shows different impact factor thresholds; for instance, a journal in the general dentistry (*Journal of Dental Research*) field might have an impact factor up to 40 times as high as the corresponding figure in the oral surgery category. The ES is acquiring traction as it highlights the influence of specific papers, but dependence entirely on citation count still limits it. The remaining bibliometrics are less well-known as predictors of citations; for instance, the number of publications at least scales with a journal size; however, it does not consider quality; this left the ES as a victor in an unbalanced contest [99].

BMPs have numerous applications in the medical field including anterior [100–102], posterior [103], and transforaminal lumbar interbody fusions [104, 105]; anterior cervical discectomy and fusion [106]; posterolateral fusion [107, 108]; open tibia fractures [109–111]; and segmental defects [112]. In oral and maxillofacial surgery, BMPs are used for alveolar bone regeneration [8, 13–15, 17, 30, 58], sinus lift augmentation [3, 5, 31, 61, 113], dental implants [16, 32], and periodontal [33–37] and dental regeneration [24–29]. In the present study, the most-cited type of BMP was BMP-2. The explanations for this might be that BMP-2 is FDA approved and, when properly used, it eliminates the requirement to harvest autogenous bone for grafting procedures, benefiting both the patient and surgeon [114]. This led to it being the most widely used BMP-type worldwide.

This bibliometric study has a few limitations. First, for a particular research area, many factors may affect the citation count, including article age, journal of publication, author's reputation, institution, and country of origin as well as the original language. Second, the analysis of self-citations and citations in textbooks and lectures was not performed. Furthermore, it is a fact that some authors may be inclined to cite the articles from a specific journal in which they intend to publish an article [115]. Third, the analysis of the contributing countries and institutions was based on the address of the corresponding author. A statistical bias may occur once the address of the corresponding author is changed [116]. Furthermore, for corresponding authors working in multiple institutions, we only considered the first institution.

## 5. Conclusion and Future Direction

This bibliometric analysis revealed that the age of article and the impact factor of journal were statistically associated with citation count. Surprisingly, unlike several other bibliometric studies performed within dentistry and medicine, the number of animal studies, *in vitro* studies, and randomized controlled trails was higher than the literature review articles [41, 76, 83]. Despite the substantial advancements in the quality of research on BMPs in recent decades, no evidence level I research could secure its position among the most frequently cited papers. Although most-cited articles' list soon may include publications representing meta-analyses and systematic reviews, at the present time, this has not yet been a decisive determinant of citations pertaining to research on the applications of BMPs in dentistry.

These are exciting times in the field of BMPs, and their potent osteoinductive properties place them on the threshold of clinical applications in dentistry. There is a need for further studies to develop novel biomaterials for BMP delivery in such applications as periodontal and craniofacial applications and in dentin regeneration in teeth.

## Figures and Tables

**Table 1:** Journal impact factor, CiteScore, Eigenfactor, and other bibliometrics of the journals contributing to the top 50 most-cited articles.

Journal name	Self-citations	Rank	Highest percentile	Citations (2016-2019)	Documents (2016-2019)	% cited	5-year JIF^∗^	CiteScore	Eigenfactor Score	No. of articles
J Periodontol	111	3/11	77%	3384	650	81	3.614	5.2	0.013	9
Clin Oral Implants Res	231	1/48	98%	5632	753	85	4.044	7.5	0.017	9
J Dent Res	187	1/113	99%	6530	727	84	5.844	9.0	0.023	7
J Clin Periodontol	315	2/11	86%	4387	592	81	5.213	7.4	0.014	4
J Oral Maxillofac Surg	246	14/48	70%	4420	1584	65	2.020	2.8	0.017	3
J Craniofac Surg	452	—	—	—	—	—	1.050	—	0.012	3
Int J Periodont Rest Dent	39	20/48	59%	1016	471	61	1.739	2.2	0.003	3
Eur J Oral Sci	10	21/113	81%	928	281	70	2.225	3.3	0.004	3
Arch Oral Biol	100	20/113	82%	3492	1031	75	2.112	3.4	0.007	3
Int J Oral Maxillofac Implants	160	10/48	80%	2294	681	72	2.987	3.4	0.010	2
J Endod	605	4/113	96%	6884	1110	84	3.380	6.2	0.016	1
J Craniomaxillofac Surg	205	11/48	78%	3836	1163	67	2.169	3.3	0.007	1
Int J Oral Maxillofac Surg	165	7/48	86%	3385	913	74	2.392	3.7	0.010	1
Clin Oral Investig	229	10/113	91%	5918	1350	80	2.710	4.4	0.009	1

^∗^Data only since 2015.

**Table 2:** Simple linear regression analysis of different journal metrics.

Variables	Coefficients standard error	Standardized coefficient beta	*p* value	95% CI Lower bound upper bound	
Self-citation	2.310	0.122	0.025^∗^	0.914	11.208
Highest percentile	25.145	0.058	0.714	-54.875	74.402
Documents	2.174	0.585	0.301	1.608	4.483
Cited percentage	39.689	0.656	0.709	-86.320	117.728
5-year JIF	385.587	-0.277	0.331	-1405.894	576.471
CiteScore	303.866	0.962	0.036^∗^	81.244	1643.468
Eigenfactor Score	59268.159	0.530	0.015^∗^	42682.078	306797.453

^∗^Statistically significant (*p* value less than 0.05). CI: confidence interval; JIF: journal impact factor.

**Table 3:** Distribution and study designs and type of BMP across the 50 most-cited articles.

Type of BMP	BMP-2	BMP-4	BMP-7	BMP-12	Others
Animal study	24	2	3	—	1
Clinical trial	7	—	1	—	—
*In vitro* study	2	—	2	1	—
Randomized controlled trial	4	—	—	—	—
Case series	2	—	—	—	—
Literature review	—	—	—	—	1

## Supplementary Materials

Supplementary 1 Supplementary Table 1: Ranking list of the top 50 most cited articles.

Supplementary 2 Supplementary Figure 1: Illustrative depiction of article selection process.

## References

[1] Urist M. R., Strates B. S. (2016). Bone morphogenetic protein. *Journal of Dental Research*.

[2] Canalis E., Economides A. N., Gazzerro E. (2003). Bone morphogenetic proteins, their antagonists, and the skeleton. *Endocrine Reviews*.

[3] Koch F. P., Becker J., Terheyden H., Capsius B., Wagner W., on behalf of the research group of this multicenter clinical trial (2010). A prospective, randomized pilot study on the safety and efficacy of recombinant human growth and differentiation factor-5 coated onto *β*-tricalcium phosphate for sinus lift augmentation. *Clinical Oral Implants Research*.

[4] Susin C., Qahash M., Polimeni G. (2010). Alveolar ridge augmentation using implants coated with recombinant human bone morphogenetic protein-7 (rhBMP-7/rhOP-1): histological observations. *Journal of Clinical Periodontology*.

[5] Gruber R. M., Ludwig A., Merten H. A., Pippig S., Kramer F. J., Schliephake H. (2009). Sinus floor augmentation with recombinant human growth and differentiation factor-5 (rhGDF-5): a pilot study in the Goettingen miniature pig comparing autogenous bone and rhGDF-5. *Clinical Oral Implants Research*.

[6] Leknes K. N., Yang J., Qahash M., Polimeni G., Susin C., Wikesjö U. M. E. (2008). Alveolar ridge augmentation using implants coated with recombinant human bone morphogenetic protein-7 (rhBMP-7/rhOP-1): radiographic observations. *Journal of Clinical Periodontology*.

[7] al-Salleeh F., Beatty M. W., Reinhardt R. A., Petro T. M., Crouch L. (2008). Human osteogenic protein-1 induces osteogenic differentiation of adipose-derived stem cells harvested from mice. *Archives of Oral Biology*.

[8] Clokie C. M., Sándor G. K. (2008). Reconstruction of 10 major mandibular defects using bioimplants containing BMP-7. *Journal of the Canadian Dental Association*.

[9] Casagrande L., Demarco F. F., Zhang Z., Araujo F. B., Shi S., Nör J. E. (2010). Dentin-derived BMP-2 and odontoblast differentiation. *Journal of Dental Research*.

[10] Zhong W. J., Zhang W. B., Ma J. Q., Wang H., Pan Y. C., Wang L. (2011). Periostin-like-factor-induced bone formation within orthopedic maxillary expansion. *Orthodontics & Craniofacial Research*.

[11] Wise G. E., He H., Gutierrez D. L., Ring S., Yao S. (2011). Requirement of alveolar bone formation for eruption of rat molars. *European Journal of Oral Sciences*.

[12] Kroczek A., Park J., Birkholz T., Neukam F. W., Wiltfang J., Kessler P. (2010). Effects of osteoinduction on bone regeneration in distraction: results of a pilot study. *Journal of Cranio-Maxillofacial Surgery*.

[13] Schwarz F., Ferrari D., Sager M., Herten M., Hartig B., Becker J. (2009). Guided bone regeneration using rhGDF-5- and rhBMP-2-coated natural bone mineral in rat calvarial defects. *Clinical Oral Implants Research*.

[14] Dupoirieux L., Pohl J., Hanke M., Pourquier D. (2009). A preliminary report on the effect of dimeric rhGDF-5 and its monomeric form rhGDF-5C465A on bone healing of rat cranial defects. *Journal of Cranio-Maxillofacial Surgery*.

[15] Trombelli L., Farina R., Marzola A., Bozzi L., Liljenberg B., Lindhe J. (2008). Modeling and remodeling of human extraction sockets. *Journal of Clinical Periodontology*.

[16] Palmieri A., Pezzetti F., Brunelli G. (2008). Short-period effects of zirconia and titanium on osteoblast microRNAs. *Clinical Implant Dentistry and Related Research*.

[17] Ayoub A., Challa S. R. R., Abu-Serriah M. (2007). Use of a composite pedicled muscle flap and rhBMP-7 for mandibular reconstruction. *International Journal of Oral and Maxillofacial Surgery*.

[18] Ho S., Peel S. A., Hu Z. M., Sándor G. K., Clokie C. M. (2010). Augmentation of the maxillary sinus: comparison of bioimplants containing bone morphogenetic protein and autogenous bone in a rabbit model. *Journal of the Canadian Dental Association*.

[19] Wozney J. M., Rosen V. (1998). Bone morphogenetic protein and bone morphogenetic protein gene family in bone formation and repair. *Clinical Orthopaedics and Related Research*.

[20] McKay B., Sandhu H. S. (2002). Use of recombinant human bone morphogenetic protein-2 in spinal fusion applications. *Spine*.

[21] Orth M., Kruse N. J., Braun B. J. (2017). BMP-2-coated mineral coated microparticles improve bone repair in atrophic non-unions. *European Cells & Materials*.

[22] Jain A., Kumar S., Aggarwal A. N., Jajodia N. (2015). Augmentation of bone healing in delayed and atrophic nonunion of fractures of long bones by partially decalcified bone allograft (decal bone). *Indian journal of orthopaedics*.

[23] Carragee E. J., Hurwitz E. L., Weiner B. K. (2011). A critical review of recombinant human bone morphogenetic protein-2 trials in spinal surgery: emerging safety concerns and lessons learned. *The Spine Journal*.

[24] Furey A., Hjelmhaug J., Lobner D. (2010). Toxicity of flow line, Durafill VS, and Dycal to dental pulp cells: effects of growth factors. *Journal of Endodontics*.

[25] da Silva B., Assed L., de Paula e Silva G., Wanderley F., Leonardo M. R., Assed S. (2008). Radiographic evaluation of pulpal and periapical response of dogs' teeth after pulpotomy and use of recombinant human bone morphogenetic protein-7 as a capping agent. *Journal of Dentistry for Children*.

[26] da Silva L. A. B., de Paula e Silva F. W., Leonardo M. R., Assed S. (2007). Pulpal and periapical response of dogs' teeth after pulpotomy and use of recombinant human bone morphogenetic protein-7 as a capping agent. *Journal of Dentistry for Children*.

[27] Narukawa M., Suzuki N., Takayama T., Shoji T., Otsuka K., Ito K. (2007). Enamel matrix derivative stimulates chondrogenic differentiation of ATDC5 cells. *Journal of Periodontal Research*.

[28] Lin Z.-M., Qin W., Zhang N. H., Xiao L., Ling J. Q. (2007). Adenovirus-mediated recombinant human bone morphogenetic protein-7 expression promotes differentiation of human dental pulp cells. *Journal of Endodontics*.

[29] Cabrera S., Barden D., Wolf M., Lobner D. (2007). Effects of growth factors on dental pulp cell sensitivity to amalgam toxicity. *Dental Materials*.

[30] Mhawi A. A., Peel S. A. F., Fok T. C. O., Clokie C. M. L. (2007). Bone regeneration in athymic calvarial defects with Accell DBM100. *Journal of Craniofacial Surgery*.

[31] Iglesias-Linares A., Yañez-Vico R. M., Moreno-Fernandez A. M., Mendoza-Mendoza A., Solano-Reina E. (2012). Corticotomy-assisted orthodontic enhancement by bone morphogenetic protein-2 administration. *Journal of Oral and Maxillofacial Surgery*.

[32] Polimeni G., Wikesjö U. M., Susin C. (2010). Alveolar ridge augmentation using implants coated with recombinant human growth/differentiation factor-5: histologic observations. *Journal of Clinical Periodontology*.

[33] Herberg S., Siedler M., Pippig S. (2008). Development of an injectable composite as a carrier for growth factor-enhanced periodontal regeneration. *Journal of Clinical Periodontology*.

[34] Nakamura T., Yamamoto M., Tamura M., Izumi Y. (2003). Effects of growth/differentiation factor-5 on human periodontal ligament cells. *Journal of Periodontal Research*.

[35] Min C. K., Wikesjö U. M. E., Park J. C. (2011). Wound healing/regeneration using recombinant human growth/differentiation factor-5 in an injectable poly-lactide-co-glycolide-acid composite carrier and a one-wall intra-bony defect model in dogs. *Journal of Clinical Periodontology*.

[36] Kwon D. H., Bennett W., Herberg S. (2010). Evaluation of an injectable rhGDF-5/PLGA construct for minimally invasive periodontal regenerative procedures: a histological study in the dog. *Journal of Clinical Periodontology*.

[37] Kwon D. H., Bisch F. C., Herold R. W. (2010). Periodontal wound healing/regeneration following the application of rhGDF-5 in a *β*-TCP/PLGA carrier in critical-size supra-alveolar periodontal defects in dogs. *Journal of Clinical Periodontology*.

[38] Hirsch J. E. (2005). An index to quantify an individual's scientific research output. *Proceedings of the National Academy of Sciences of the United States of America*.

[39] Hennessey K., Afshar K., Macneily A. E. (2009). The top 100 cited articles in urology. *Canadian Urological Association Journal*.

[40] Ponce F. A., Lozano A. M. (2010). Highly cited works in neurosurgery. Part I: the 100 top-cited papers in neurosurgical journals. *Journal of Neurosurgery*.

[41] Lefaivre K. A., Shadgan B., O’Brien P. J. (2011). 100 most cited articles in orthopaedic surgery. *Clinical Orthopaedics and Related Research*.

[42] Stack S. (2012). Citation classics in suicide and life threatening behavior: a research note. *Suicide and Life-threatening Behavior*.

[43] Ruttenstock E., Friedmacher F., Höllwarth M. E., Coran A. G., Puri P. (2012). The 100 most-cited articles in pediatric surgery international. *Pediatric Surgery International*.

[44] Feijoo J. F., Limeres J., Fernández-Varela M., Ramos I., Diz P. (2014). The 100 most cited articles in dentistry. *Clinical Oral Investigations*.

[45] Fardi A., Kodonas K., Gogos C., Economides N. (2011). Top-cited articles in endodontic journals. *Journal of Endodontics*.

[46] Tarazona B., Lucas-Dominguez R., Paredes-Gallardo V., Alonso-Arroyo A., Vidal-Infer A. (2018). The 100 most-cited articles in orthodontics: a bibliometric study. *The Angle Orthodontist*.

[47] Corbella S., Francetti L., Taschieri S., Weinstein R., del Fabbro M. (2017). Analysis of the 100 most-cited articles in periodontology. *Journal of Investigative and Clinical Dentistry*.

[48] Praveen G., Chaithanya R., Alla R. K., Shammas M., Abdurahiman V. T., Anitha A. (2020). The 100 most cited articles in prosthodontic journals: a bibliometric analysis of articles published between 1951 and 2019. *The Journal of Prosthetic Dentistry*.

[49] Zhang Q., Yue Y., Shi B., Yuan Z. (2018). A bibliometric analysis of cleft lip and palate-related publication trends from 2000 to 2017. *The Cleft Palate-Craniofacial Journal*.

[50] Aslam-Pervez N., Lubek J. E. (2018). Most cited publications in oral and maxillofacial surgery: a bibliometric analysis. *Oral and Maxillofacial Surgery*.

[51] Gondivkar S. M., Sarode S. C., Gadbail A. R., Gondivkar R. S., Chole R., Sarode G. S. (2018). Bibliometric analysis of 100 most cited articles on oral submucous fibrosis. *Journal of Oral Pathology & Medicine*.

[52] Andersen J., Belmont J., Cho C. T. (2006). Journal impact factor in the era of expanding literature. *Journal of Microbiology Immunology and Infection*.

[53] Shamseer L., Moher D., Clarke M. (2015). Preferred reporting items for systematic review and meta-analysis protocols (PRISMA-P) 2015: elaboration and explanation. *BMJ*.

[54] Ahmad P., Alam M. K., Jakubovics N. S., Schwendicke F., Asif J. A. (2019). 100 years of the Journal of Dental Research: a bibliometric analysis. *Journal of Dental Research*.

[55] Li H., Zhao X., Zheng P. (2015). Classic citations in main primary health care journals: a PRISMA-compliant systematic literature review and bibliometric analysis. *Medicine*.

[56] Roldan-Valadez E., Salazar-Ruiz S. Y., Ibarra-Contreras R., Rios C. (2019). Current concepts on bibliometrics: a brief review about impact factor, Eigenfactor score, CiteScore, SCImago Journal Rank, Source-Normalised Impact per Paper, H-index, and alternative metrics. *Irish Journal of Medical Science*.

[57] Van Eck N. J., Waltman L. (2010). Software survey: VOSviewer, a computer program for bibliometric mapping. *Scientometrics*.

[58] Fiorellini J. P., Howell T. H., Cochran D. (2005). Randomized study evaluating recombinant human bone morphogenetic protein-2 for extraction socket augmentation. *Journal of Periodontology*.

[59] Iohara K., Nakashima M., Ito M., Ishikawa M., Nakasima A., Akamine A. (2004). Dentin regeneration by dental pulp stem cell therapy with recombinant human bone morphogenetic protein 2. *Journal of Dental Research*.

[60] Jung R. E., Glauser R., Schärer P., Hämmerle C. H. F., Sailer H. F., Weber F. E. (2003). Effect of rhBMP-2 on guided bone regeneration in humans. *Clinical Oral Implants Research*.

[61] Boyne P. J., Lilly L. C., Marx R. E. (2005). De novo bone induction by recombinant human bone morphogenetic protein-2 (rhBMP-2) in maxillary sinus floor augmentation. *Journal of Oral and Maxillofacial Surgery*.

[62] Triplett R. G., Nevins M., Marx R. E. (2009). Pivotal, randomized, parallel evaluation of recombinant human bone morphogenetic protein-2/absorbable collagen sponge and autogenous bone graft for maxillary sinus floor augmentation. *Journal of Oral and Maxillofacial Surgery*.

[63] Cicciù M., Scott A., Cicciù D., Tandon R., Maiorana C. (2014). Recombinant human bone morphogenetic protein-2 promote and stabilize hard and soft tissue healing for large mandibular new bone reconstruction defects. *Journal of Craniofacial Surgery*.

[64] Fardi A., Kodonas K., Lillis T., Veis A. (2017). Top-cited articles in implant dentistry. *International Journal of Oral & Maxillofacial Implants*.

[65] Ahmad P., Dummer P. M. H., Noorani T. Y., Asif J. A. (2019). The top 50 most-cited articles published in the International Endodontic Journal. *International Endodontic Journal*.

[66] Jafarzadeh H., Sarraf Shirazi A., Andersson L. (2015). The most-cited articles in dental, oral, and maxillofacial traumatology during 64 years. *Dental Traumatology*.

[67] Yılmaz B., Dinçol M. E., Yalçın T. Y. (2019). A bibliometric analysis of the 103 top-cited articles in endodontics. *Acta Odontologica Scandinavica*.

[68] Kulkarni A. V., Aziz B., Shams I., Busse J. W. (2009). Comparisons of citations in Web of Science, Scopus, and Google Scholar for articles published in general medical journals. *JAMA*.

[69] Bakkalbasi N., Bauer K., Glover J., Wang L. (2006). Three options for citation tracking: Google Scholar, Scopus and Web of Science. *Biomedical Digital Libraries*.

[70] Falagas M. E., Pitsouni E. I., Malietzis G. A., Pappas G. (2008). Comparison of PubMed, Scopus, Web of Science, and Google Scholar: strengths and weaknesses. *The FASEB Journal*.

[71] Harzing A.-W. K., Van der Wal R. (2008). Google Scholar as a new source for citation analysis. *Ethics in science and environmental politics*.

[72] Lin C. L., Ho Y.-S. (2015). A bibliometric analysis of publications on pluripotent stem cell research. *Cell Journal*.

[73] Shuaib W., Khan M. S., Shahid H., Valdes E. A., Alweis R. (2015). Bibliometric analysis of the top 100 cited cardiovascular articles. *The American Journal of Cardiology*.

[74] Chu T., Kwok H. T., Chan J., Tse F. Y. F. (2019). The 100 most cited manuscripts in head and neck cancer: a bibliometric analysis. *The Journal of Laryngology & Otology*.

[75] Tam W. W. S., Wong E. L. Y., Wong F. C. Y., Hui D. S. C. (2013). Citation classics: top 50 cited articles in ‘respiratory system’. *Respirology*.

[76] Baltussen A., Kindler C. H. (2004). *Citation classics in anesthetic journals*. *Anesthesia & Analgesia*.

[77] Ugolini D., Neri M., Cesario A. (2012). Scientific production in cancer rehabilitation grows higher: a bibliometric analysis. *Supportive Care in Cancer*.

[78] Jayaratne Y. S. N., Zwahlen R. A. (2015). The evolution of dental journals from 2003 to 2012: a bibliometric analysis. *PLoS One*.

[79] Geminiani A., Ercoli C., Feng C., Caton J. G. (2014). Bibliometrics study on authorship trends in periodontal literature from 1995 to 2010. *Journal of Periodontology*.

[80] Tarazona B., Vidal-Infer A., Alonso-Arroyo A. (2017). Bibliometric analysis of the scientific production in implantology (2009–2013). *Clinical Oral Implants Research*.

[81] Khan M. S., Usman M. S., Fatima K. (2017). Characteristics of highly cited articles in interventional cardiology. *The American Journal of Cardiology*.

[82] Bueno-Aguilera F., Jimenez-Contreras E., Lucena-Martin C., Pulgar-Encinas R. (2016). Dental research in Spain. A bibliometric analysis on subjects, authors and institutions (1993-2012). *Medicina oral, patologia oral y cirugia bucal*.

[83] Paladugu R., Schein M., Gardezi S., Wise L. (2002). One hundred citation classics in general surgical journals. *World Journal of Surgery*.

[84] Loonen M. P., Hage J. J., Kon M. (2008). Plastic surgery classics: characteristics of 50 top-cited articles in four plastic surgery journals since 1946. *Plastic and Reconstructive Surgery*.

[85] Brandt J. S., Downing A. C., Howard D. L., Kofinas J. D., Chasen S. T. (2010). Citation classics in obstetrics and gynecology: the 100 most frequently cited journal articles in the last 50 years. *American Journal of Obstetrics and Gynecology*.

[86] Lefaivre K. A., Guy P., OʼBrien P. J., Blachut P. A., Shadgan B., Broekhuyse H. M. (2010). Leading 20 at 20: top cited articles and authors in the Journal of Orthopaedic Trauma, 1987-2007. *Journal of Orthopaedic Trauma*.

[87] Shadgan B., Roig M., HajGhanbari B., Reid W. D. (2010). *Top-cited articles in rehabilitation*. *Archives of Physical Medicine and Rehabilitation*.

[88] Arshad A. I., Ahmad P., Dummer P. M. H. (2020). Citation classics on dental caries: a systematic review. *European Journal of Dentistry*.

[89] Campbell F. M. (1990). National bias: a comparison of citation practices by health professionals. *Bulletin of the Medical Library Association*.

[90] Baltussen A., Kindler C. H. (2004). Citation classics in critical care medicine. *Intensive Care Medicine*.

[91] Fenton J., Roy D., Hughes J. P., Jones A. S. (2002). A century of citation classics in otolaryngology—head and neck surgery journals. *The Journal of Laryngology & Otology*.

[92] Jung R. E., Windisch S. I., Eggenschwiler A. M., Thoma D. S., Weber F. E., Hämmerle C. H. F. (2009). A randomized-controlled clinical trial evaluating clinical and radiological outcomes after 3 and 5 years of dental implants placed in bone regenerated by means of GBR techniques with or without the addition of BMP-2. *Clinical Oral Implants Research*.

[93] Badyal D. K., Desai C. (2014). Animal use in pharmacology education and research: the changing scenario. *Indian journal of pharmacology*.

[94] Brinjikji W., Klunder A., Kallmes D. F. (2013). The 100 most-cited articles in the imaging literature. *Radiology*.

[95] Shuaib W., Acevedo J. N., Khan M. S., Santiago L. J., Gaeta T. J. (2015). The top 100 cited articles published in emergency medicine journals. *The American Journal of Emergency Medicine*.

[96] Shuaib W., Costa J. L. (2015). Anatomy of success: 100 most cited articles in diabetes research. *Therapeutic Advances in Endocrinology and Metabolism*.

[97] Usman M. S., Siddiqi T. J., Khan M. S. (2017). A scientific analysis of the 100 citation classics of valvular heart disease. *The American Journal of Cardiology*.

[98] Huo Y.-Q., Pan X. H., Li Q. B. (2015). Fifty top-cited classic papers in orthopedic elbow surgery: a bibliometric analysis. *International Journal of Surgery*.

[99] Roldan-Valadez E., Orbe-Arteaga U., Rios C. (2018). Eigenfactor score and alternative bibliometrics surpass the impact factor in a 2-years ahead annual-citation calculation: a linear mixed design model analysis of radiology, nuclear medicine and medical imaging journals. *La Radiologia Medica*.

[100] Burkus J. K., Sandhu H. S., Gornet M. F., Longley M. C. (2005). Use of rhBMP-2 in combination with structural cortical allografts: clinical and radiographic outcomes in anterior lumbar spinal surgery. *The Journal of Bone and Joint Surgery (American)*.

[101] Burkus J. K., Heim S. E., Gornet M. F., Zdeblick T. A. (2003). Is INFUSE bone graft superior to autograft bone? An integrated analysis of clinical trials using the LT-CAGE lumbar tapered fusion device. *Journal of Spinal Disorders & Techniques*.

[102] Burkus J. K., Gornet M. F., Dickman C. A., Zdeblick T. A. (2002). Anterior lumbar interbody fusion using rhBMP-2 with tapered interbody cages. *Journal of Spinal Disorders & Techniques*.

[103] Haid R. W., Branch C. L., Alexander J. T., Burkus J. K. (2004). Posterior lumbar interbody fusion using recombinant human bone morphogenetic protein type 2 with cylindrical interbody cages. *The Spine Journal*.

[104] Mummaneni P. V., Pan J., Haid R. W., Rodts G. E. (2004). Contribution of recombinant human bone morphogenetic protein—2 to the rapid creation of interbody fusion when used in transforaminal lumbar interbody fusion: a preliminary report. invited submission from the Joint Section Meeting on Disorders of the Spine and Peripheral Nerves, March 2004. *Journal of Neurosurgery: Spine*.

[105] Schwender J. D., Holly L. T., Rouben D. P., Foley K. T. (2005). Minimally invasive transforaminal lumbar interbody fusion (TLIF). *Clinical Spine Surgery*.

[106] Baskin D. S., Ryan P., Sonntag V., Westmark R., Widmayer M. A. (2003). A prospective, randomized, controlled cervical fusion study using recombinant human bone morphogenetic protein-2 with the CORNERSTONE-SR™ allograft ring and the ATLANTIS™ anterior cervical plate. *Spine*.

[107] Glassman S. D., Carreon L., Djurasovic M. (2007). Posterolateral lumbar spine fusion with INFUSE bone graft. *The Spine Journal*.

[108] Singh K., Smucker J. D., Gill S., Boden S. D. (2006). Use of recombinant human bone morphogenetic protein-2 as an adjunct in posterolateral lumbar spine fusion. *Journal of Spinal Disorders & Techniques*.

[109] Swiontkowski M. F., Aro H. T., Donell S. (2006). Recombinant human bone morphogenetic protein-2 in open tibial fractures. *The Journal of Bone and Joint Surgery-American Volume*.

[110] Jones A. L., Bucholz R. W., Bosse M. J. (2006). Recombinant human BMP-2 and allograft compared with autogenous bone graft for reconstruction of diaphyseal tibial fractures with cortical defects. *The Journal of Bone and Joint Surgery-American Volume*.

[111] Govender S., Csimma C., Genant H. K. (2002). Recombinant human bone morphogenetic protein-2 for treatment of open tibial fractures. *The Journal of Bone and Joint Surgery-American Volume*.

[112] Schwartz N., Hicks B. (2006). Segmental bone defects treated using recombinant human bone morphogenetic protein. *Journal of Orthopaedics*.

[113] Boyne P. J., Marx R. E., Nevins M. (1997). A feasibility study evaluating rhBMP-2/absorbable collagen sponge for maxillary sinus floor augmentation. *International journal of periodontics & restorative dentistry*.

[114] McKay W. F., Peckham S. M., Badura J. M. (2007). A comprehensive clinical review of recombinant human bone morphogenetic protein-2 (INFUSE® Bone Graft). *International Orthopaedics*.

[115] Zhao X., Guo L., Lin Y. (2016). The top 100 most cited scientific reports focused on diabetes research. *Acta Diabetologica*.

[116] Zhang Y., Huang J., Du L. (2017). The top-cited systematic reviews/meta-analyses in tuberculosis research: a PRISMA-compliant systematic literature review and bibliometric analysis. *Medicine*.

