# Supplementary material for: Applications of Bone Morphogenetic Proteins in Dentistry: A Bibliometric Analysis
Source: Biomed Res Int. 2020 Oct 24;2020:5971268. doi: 10.1155/2020/5971268 (PMC7604587; doi:10.1155/2020/5971268)
Supplement: Supplementary 1 — Supplementary Table 1: Ranking list of the top 50 most cited articles. [file 5971268.f1.docx]

**Supplementary Table 1**. Ranking list of the top 50 most cited articles

| **Sr No.** | **Title of the article** | **Citation count (WoS)** | **Citation count (GS)** | **Citation density** | **Current citation index (2019)** |
| --- | --- | --- | --- | --- | --- |
| 1 | Urist MR, Strates BS. Bone morphogenetic proteins. *Journal of Dental Research*. 1971:50;1392-1406. | 557 | 1080 | 11.60 | 15 |
| 2 | Fiorellini JP, Howell TH, Cochran D, Malmquist J, Lilly LC, Spagnoli D, Toljanic J, Jones A, Nevins M. Randomized study evaluating recombinant human bone morphogenetic protein-2 for extraction socket augmentation. *Journal of Periodontology*. 2005;76:605-613 | 270 | 432 | 19.29 | 22 |
| 3 | Iohara K, Nakashima M, Ito M, Ishikawa M, Nakasima A, Akamine A. Dentin regeneration by dental pulp stem cell therapy with recombinant human bone morphogenetic protein 2*. Journal of dental research*. 2004;83:590-595. | 261 | 446 | 17.40 | 11 |
| 4 | Boyne PJ, Lilly LC, Marx RE, Moy PK, Nevins M, Spagnoli DB, Triplett RG. De novo bone induction by recombinant human bone morphogenetic protein-2 (rhBMP-2) in maxillary sinus floor augmentation*. Journal of Oral and Maxillofacial Surgery*. 2005;63:1693-1707. | 253 | 416 | 18.07 | 11 |
| 5 | Sigurdsson TJ, Lee MB, Kubota K, Turek TJ, Wozney JM, Wikesjö UM. Periodontal repair in dogs: recombinant human bone morphogenetic protein‐2 significantly enhances periodontal regeneration*. Journal of periodontology*. 1995;66:131-138. | 242 | 405 | 10.08 | 2 |
| 6 | Jung RE, Glauser R, Schärer P, Hämmerle CH, Sailer HF, Weber FE. Effect of rhBMP‐2 on guided bone regeneration in humans: A randomized, controlled clinical and histomorphometric study*. Clinical oral implants research*. 2003;14:556-568. | 200 | 314 | 12.50 | 16 |
| 7 | Howell TH, Fiorellini J, Jones A, Alder M, Nummikoski P, Lazaro M, Lilly L, Cochran D. A feasibility study evaluating rhBMP-2/absorbable collagen sponge device for local alveolar ridge preservation or augmentation*. International journal of periodontics & restorative dentistry*. 1997;17. | 199 | 407 | 9.05 | 7 |
| 8 | Nakashima M. Induction of dentin formation on canine amputated pulp by recombinant human bone morphogenetic proteins (BMP)-2 and-4*. Journal of Dental Research*. 1994;73:1515-1522. | 187 | 323 | 7.48 | 6 |
| 9 | Triplett RG, Nevins M, Marx RE, Spagnoli DB, Oates TW, Moy PK, Boyne PJ. Pivotal, randomized, parallel evaluation of recombinant human bone morphogenetic protein-2/absorbable collagen sponge and autogenous bone graft for maxillary sinus floor augmentation*. Journal of Oral and Maxillofacial Surgery*. 2009;67:1947-1960. | 176 | 272 | 17.60 | 11 |
| 10 | King G, King N, Cruchley A, Wozney J, Hughes F. Recombinant human bone morphogenetic protein-2 promotes wound healing in rat periodontal fenestration defects*. Journal of dental research*. 1997;76:1460-1470. | 168 | 263 | 7.64 | 5 |
| 11 | Herford AS, Boyne PJ. Reconstruction of mandibular continuity defects with bone morphogenetic protein-2 (rhBMP-2)*. Journal of Oral and Maxillofacial Surgery*. 2008;66:616-624. | 164 | 285 | 14.91 | 9 |
| 12 | Cochran DL, Schenk R, Buser D, Wozney JM, Jones AA. Recombinant human bone morphogenetic protein‐2 stimulation of bone formation around endosseous dental implants*. Journal of periodontology*. 1999;70:139-150. | 160 | 260 | 8.00 | 3 |
| 13 | Cochran DL, Jones AA, Lilly LC, Fiorellini JP, Howell H. Evaluation of recombinant human bone morphogenetic protein‐2 in oral applications including the use of endosseous implants: 3‐year results of a pilot study in humans*. Journal of Periodontology*. 2000;71:1241-1257. | 146 | 251 | 7.68 | 4 |
| 14 | Nevins M, Kirker-Head C, Nevins M, Wozney JA, Palmer R, Graham D. Bone formation in the goat maxillary sinus induced by absorbable collagen sponge implants impregnated with recombinant human bone morphogenetic protein-2*. International Journal of Periodontics & Restorative Dentistry*. 1996;16. | 141 | 223 | 6.13 | 1 |
| 15 | Wozney JM. The bone morphogenetic protein family: multifunctional cellular regulators in the embryo and adult*. European Journal of Oral Sciences*. 1998;106:160-166. | 140 | 253 | 6.67 | 0 |
| 16 | Wikesjö UM, Guglielmoni P, Promsudthi A, Cho KS, Trombelli L, Selvig KA, Jin L, Wozney JM. Periodontal repair in dogs: effect of rhBMP‐2 concentration on regeneration of alveolar bone and periodontal attachment*. Journal of clinical periodontology*. 1999;26:392-400. | 140 | 259 | 7.00 | 2 |
| 17 | Kinoshita A, Oda S, Takahashi K, Yokota S, Ishikawa I. Periodontal regeneration by application of recombinant human bone morphogenetic protein‐2 to horizontal circumferential defects created by experimental periodontitis in beagle dogs*. Journal of periodontology*. 1997;68:103-109. | 139 | 221 | 6.32 | 3 |
| 18 | Jin QM, Anusaksathien O, Webb S, Rutherford R, Giannobile W. Gene therapy of bone morphogenetic protein for periodontal tissue engineering*. Journal of periodontology*. 2003;74:202-213. | 138 | 236 | 8.63 | 2 |
| 19 | Casagrande L, Demarco F, Zhang Z, Araujo F, Shi S, Nör J. Dentin-derived BMP-2 and odontoblast differentiation*. Journal of dental research*. 2010;89:603-608. | 138 | 247 | 15.33 | 6 |
| 20 | Sigurdsson TJ, Fu E, Tatakis DN, Rohrer MD, Wikesjö UM. Bone morphogenetic protein‐2 for peri‐implant bone regeneration and osseointegration*. Clinical Oral Implants Research*. 1997;8:367-374. | 136 | 207 | 6.18 | 2 |
| 21 | Boyne PJ, Marx RE, Nevins M, Triplett G, Lazaro E, Lilly LC, Alder M, Nummikoski P. A feasibility study evaluating rhBMP-2/absorbable collagen sponge for maxillary sinus floor augmentation*. International journal of periodontics & restorative dentistry*. 1997;17. | 124 | 627 | 5.64 | 1 |
| 22 | Hanisch O, Tatakis DN, Boskovic MM, Rohrer MD, Wikesjö UM. Bone formation and reosseointegration in peri-implantitis defects following surgical implantation of rhBMP-2*. International Journal of Oral & Maxillofacial Implants*. 1997;12. | 121 | 220 | 5.50 | 2 |
| 23 | Nakashima M. Induction of dentine in amputated pulp of dogs by recombinant human bone morphogenetic proteins-2 and 4 with collagen matrix. *Archives of Oral Biology*. 1994;39:1085-1089. | 121 | 206 | 4.84 | 4 |
| 24 | Wikesjö UM, Qahash M, Polimeni G, Susin C, Shanaman RH, Rohrer MD, Wozney JM, Hall J. Alveolar ridge augmentation using implants coated with recombinant human bone morphogenetic protein‐2: Histologic observations*. Journal of clinical periodontology*. 2008;35:1001-1010. | 118 | 180 | 10.73 | 2 |
| 25 | Choi SH, Kim CK, Cho KS, Huh JS, Sorensen RG, Wozney JM, Wikesjö UM. Effect of recombinant human bone morphogenetic protein‐2/absorbable collagen sponge (rhBMP‐2/ACS) on healing in 3‐wall intrabony defects in dogs*. Journal of periodontology*. 2002;73:63-72. | 118 | 220 | 6.94 | 4 |
| 26 | Hanisch O, Tatakis DN, Rohrer MD, Wöhrle PS, Wozney JM, Wikesjö UM. Bone formation and osseointegration stimulated by rhBMP-2 following subantral augmentation procedures in nonhuman primates*. International Journal of Oral & Maxillofacial Implants*. 1997;12. | 109 | 161 | 4.95 | 0 |
| 27 | Chin M, Ng T, Tom WK, Carstens M. Repair of alveolar clefts with recombinant human bone morphogenetic protein (rhBMP-2) in patients with clefts*. Journal of Craniofacial Surgery*. 2005;16:778-789. | 108 | 142 | 7.71 | 5 |
| 28 | Schliephake H, Aref A, Scharnweber D, Bierbaum S, Roessler S, Sewing A. Effect of immobilized bone morphogenic protein 2 coating of titanium implants on peri‐implant bone formation*. Clinical oral implants research*. 2005;16:563-569. | 107 | 156 | 7.64 | 1 |
| 29 | Saito T, Ogawa M, Hata Y, Bessho K. Acceleration effect of human recombinant bone morphogenetic protein-2 on differentiation of human pulp cells into odontoblasts*. Journal of endodontics*. 2004;30:205-208. | 101 | 189 | 6.73 | 5 |
| 30 | Ripamonti U, Heliotis M, Rueger D, Sampath T. Induction of cementogenesis by recombinant human osteogenic protein-1 (hop-1/bmp-7) in the baboon (Papio ursinus)*. Archives of Oral Biology*. 1996;41:121-126. | 100 | 160 | 4.35 | 4 |
| 31 | Liu Y, Huse RO, de Groot K, Buser D, Hunziker EB. Delivery mode and efficacy of BMP-2 in association with implants*. Journal of dental research*. 2007;86:84-89. | 99 | 144 | 8.25 | 6 |
| 32 | Park D-J, Choi B-H, Zhu S-J, Huh J-Y, Kim B-Y, Lee S-H. Injectable bone using chitosan-alginate gel/mesenchymal stem cells/BMP-2 composites*. Journal of Cranio-Maxillofacial Surgery*. 2005;33:50-54. | 99 | 161 | 7.07 | 5 |
| 33 | Barboza EP, Duarte MEL, Geolás L, Sorensen RG, Riedel GE, Wikesjö UM. Ridge augmentation following implantation of recombinant human bone morphogenetic protein‐2 in the dog*. Journal of periodontology*. 2000;71:488-496. | 97 | 181 | 5.11 | 5 |
| 34 | Suzuki S, Nagano T, Yamakoshi Y, Gomi K, Arai T, Fukae M, Katagiri T, Oida S. Enamel matrix derivative gel stimulates signal transduction of BMP and TGF-β*. Journal of Dental Research*. 2005;84:510-514. | 94 | 145 | 6.71 | 3 |
| 35 | Wikesjö UM, Qahash M, Thomson RC, Cook AD, Rohrer MD, Wozney JM, Hardwick WR. rhBMP‐2 significantly enhances guided bone regeneration*. Clinical Oral Implants Research*. 2004;15:194-204. | 94 | 152 | 6.27 | 6 |
| 36 | Jung RE, Weber FE, Thoma DS, Ehrbar M, Cochran DL, Hämmerle CH. Bone morphogenetic protein‐2 enhances bone formation when delivered by a synthetic matrix containing hydroxyapatite/tricalciumphosphate*. Clinical oral implants research*. 2008;19:188-195. | 91 | 126 | 8.27 | 2 |
| 37 | Becker J, Kirsch A, Schwarz F, Chatzinikolaidou M, Rothamel D, Lekovic V, Jennissen HP. Bone apposition to titanium implants biocoated with recombinant human bone morphogenetic protein-2 (rhBMP-2). A pilot study in dogs*. Clinical oral investigations*. 2006;10:217-224. | 89 | 135 | 6.85 | 3 |
| 38 | Gruber R, Kandler B, Fuerst G, Fischer MB, Watzek G. Porcine sinus mucosa holds cells that respond to bone morphogenetic protein (BMP)‐6 and BMP‐7 with increased osteogenic differentiation in vitro*. Clinical Oral Implants Research*. 2004;15:575-580. | 88 | 123 | 5.87 | 4 |
| 39 | Wikesjö UM, Sorensen RG, Kinoshita A, Jian Li X, Wozney JM. Periodontal repair in dogs: effect of recombinant human bone morphogenetic protein‐12 (rhBMP‐12) on regeneration of alveolar bone and periodontal attachment: A pilot study*. Journal of clinical periodontology*. 2004;31:662-670. | 88 | 112 | 5.87 | 6 |
| 40 | Cicciù M, Scott A, Cicciù D, Tandon R, Maiorana C. Recombinant human bone morphogenetic protein-2 promote and stabilize hard and soft tissue healing for large mandibular new bone reconstruction defects*. Journal of Craniofacial Surgery*. 2014;25:860-862. | 87 | 99 | 17.40 | 11 |
| 41 | Van Den Bergh J, Ten Bruggenkate C, Groeneveld H, Burger E, Tuinzing D. Recombinant human bone morphogenetic protein‐7 in maxillary sinus floor elevation surgery in 3 patients compared to autogenous bone grafts: A clinical pilot study*. Journal of clinical periodontology*. 2000;27:627-636. | 87 | 145 | 4.58 | 3 |
| 42 | Sloan A, Rutherford R, Smith A. Stimulation of the rat dentine–pulp complex by bone morphogenetic protein-7 in vitro*. Archives of Oral Biology*. 2000;45:173-177. | 86 | 124 | 4.53 | 2 |
| 43 | Schwarz F, Rothamel D, Herten M, Ferrari D, Sager M, Becker J. Lateral ridge augmentation using particulated or block bone substitutes biocoated with rhGDF‐5 and rhBMP‐2: An immunohistochemical study in dogs*. Clinical oral implants research*. 2008;19:642-652. | 85 | 99 | 7.73 | 4 |
| 44 | Ripamonti U, Crooks J, Petit JC, Rueger DC. Periodontal tissue regeneration by combined applications of recombinant human osteogenic protein‐1 and bone morphogenetic protein‐2. A pilot study in Chacma baboons (Papio ursinus)*. European journal of oral sciences*. 2001;109:241-248. | 85 | 119 | 4.72 | 3 |
| 45 | Jung RE, Windisch SI, Eggenschwiler AM, Thoma DS, Weber FE, Hämmerle CH. A randomized‐controlled clinical trial evaluating clinical and radiological outcomes after 3 and 5 years of dental implants placed in bone regenerated by means of GBR techniques with or without the addition of BMP‐2*. Clinical oral implants research*. 2009;20:660-666. | 85 | 131 | 8.50 | 3 |
| 46 | Jovanovic SA, Hunt DR, Bernard GW, Spiekermann H, Wozney JM, Wikesjö UM. Bone reconstruction following implantation of rhBMP‐2 and guided bone regeneration in canine alveolar ridge defects*. Clinical oral implants research*. 2007;18:224-230. | 84 | 136 | 7.00 | 3 |
| 47 | Hunt DR, Jovanovic SA, Wikesjö UM, Wozney JM, Bernard GW. Hyaluronan supports recombinant human bone morphogenetic protein‐2 induced bone reconstruction of advanced alveolar ridge defects in dogs. A pilot study*. Journal of periodontology*. 2001;72:651-658. | 83 | 123 | 4.61 | 1 |
| 48 | Okubo Y, Bessho K, Fujimura K, Konishi Y, Kusumoto K, Ogawa Y, Iizuka T. Osteoinduction by recombinant human bone morphogenetic protein‐2 at intramuscular, intermuscular, subcutaneous and intrafatty sites*. International Journal of Oral & Maxillofacial Surgery*. 2000;29:62-66. | 82 | 133 | 4.32 | 1 |
| 49 | Rutherford RB, Gu K. Treatment of inflamed ferret dental pulps with recombinant bone morphogenetic protein‐7*. European journal of oral sciences*. 2000;108:202-206. | 81 | 148 | 4.26 | 3 |
| 50 | Herford AS, Tandon R, Stevens TW, Stoffella E, Cicciu M. Immediate distraction osteogenesis: the sandwich technique in combination with rhBMP-2 for anterior maxillary and mandibular defects*. Journal of Craniofacial Surgery*. 2013;24:1383-1387. | 81 | 95 | 13.50 | 12 |
