# Supplementary material for: Applications of Bone Morphogenetic Proteins in Dentistry: A Bibliometric Analysis
Source: Biomed Res Int. 2020 Oct 24;2020:5971268. doi: 10.1155/2020/5971268 (PMC7604587; doi:10.1155/2020/5971268)
Supplement: Supplementary 2 — Supplementary Figure 1: Illustrative depiction of article selection process. [file 5971268.f2.pdf]

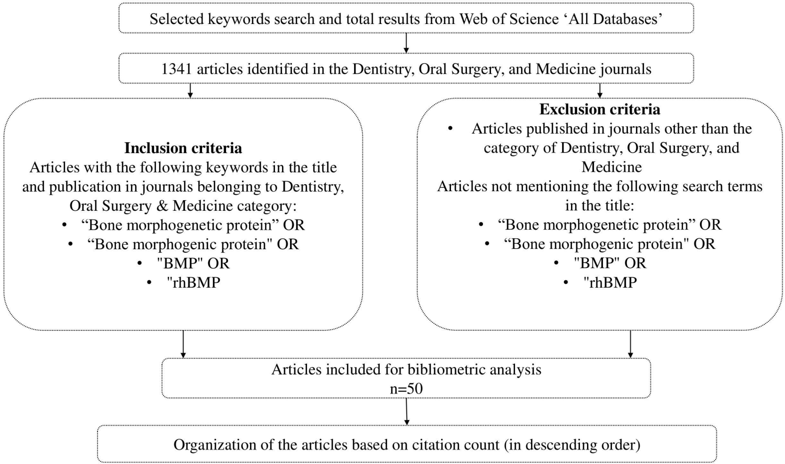
Selected keywords search and total results from Web of Science 'All Databases'
1341 articles identified in the Dentistry, Oral Surgery, and Medicine journals
Inclusion criteria
Articles with the following keywords in the title and publication in journals belonging to Dentistry, Oral Surgery & Medicine category:
• "Bone morphogenetic protein" OR
• "Bone morphogenic protein" OR
• "BMP" OR
• "rhBMP
Exclusion criteria
• Articles published in journals other than the category of Dentistry, Oral Surgery, and Medicine
Articles not mentioning the following search terms in the title:
• "Bone morphogenetic protein" OR
• "Bone morphogenic protein" OR
• "BMP" OR
• "rhBMP
Articles included for bibliometric analysis
n=50
Organization of the articles based on citation count (in descending order)
